# Improving obstetric care in low-resource settings

**DOI:** 10.1016/j.crwh.2022.e00434

**Published:** 2022-08-04

**Authors:** G. Justus Hofmeyr

**Affiliations:** a Department of Obstetrics and Gynaecology, University of Botswana, Botswana; b University of the Witwatersrand and Walter Sisulu University, South Africa

**Keywords:** Obstetric care, Low-resource settings, Global inequity, WHO labour care guide

Discrepancies in obstetric outcomes between high- and low-resource settings are among the most spectacular of global inequities, but not surprising in a world where many lack even food and water to sustain life, while others have more than they need. The proper solution, sharing of global resources, is unlikely any time soon. It goes without saying that we need both funding and the political will to provide better health services, transport, sanitation, education, housing, personal security, and so on. National governments have a responsibility to ensure that essential, affordable and life-saving commodities are available, including contraceptive methods, antibiotics, antihypertensives, magnesium sulphate, uterotonics, tocolytics, analgesics, calcium, aspirin and multiple micronutrients. Setting aside these fundamental social and political determinants of health, what can we as health care providers do here and now, with what we have?

The list of things which could improve obstetric care include both things we need to do, and things we need to stop doing.

First and foremost, we need to empower women to avoid unintended pregnancy. The all-too-common tragedy of women losing their lives in the interest of creating new life is compounded when the pregnancy was unintended. At the forefront of all obstetric care (and general medical care of women of reproductive age) should be routine counselling and offer of options to avoid unintended pregnancies, particularly low-cost options such as insertion of the copper intrauterine device shortly after giving birth, or at caesarean delivery or during miscarriage care.

We know that routine antenatal care, particularly in later pregnancy, makes a difference, because when the number of antenatal visits is reduced, more babies die [1].

We can remove artificial barriers to women accessing antenatal care, such as restricted days and times for antenatal services. We can empower women with information for self-care when they are unable to reach care, or the health services fail them: healthy food choices and what supplements are worth purchasing; warning symptoms for seeking urgent care, even outside of antenatal care appointments (bleeding, pain, excessive whole-body swelling and reduced fetal movements); and seeking access to regular blood pressure checks, particularly in late pregnancy.

For labour induction, we can choose the safer mechanical Foley catheter balloon method rather than more sophisticated and expensive prostaglandins [2].

We can implement evidence-based prevention and care for potentially life-threatening complications of pregnancy: aspirin, calcium, blood pressure screening, antihypertensives and timed delivery for hypertensive disorders; magnesium sulphate for pre-eclampsia/eclampsia; uterotonics, tranexamic acid, supportive and cause-directed treatment for postpartum haemorrhage; management of medical conditions and sepsis; corticosteroids, magnesium sulphate and delayed cord clamping for preterm births.

Labour and birth are a critical time for mothers and their babies, when unpredicted calamities often arise in even apparently ‘low-risk’ pregnancies. We can reverse policies which divert women in labour to peripheral primary care clinics, where appropriate care in the event of unanticipated complications may be long delayed. This by creating ‘primary care, midwife-led, on-site birth units’ in hospitals with obstetric care facilities [3]. For women who may be unable to access health services, we can provide birth kits [4], and misoprostol tablets for sublingual self-administration after birth.

For those who are able to reach our labour care facilities, we can promote simple practices which improve outcomes: encouraging the presence of a chosen birth companion [5]; ensuring adequate oral fluids; avoiding the supine position; and avoiding unnecessary medical interventions in the normal birth process.

Caesarean delivery is a complex issue. Many women lack access to this life-saving procedure. On the other hand, in some middle-income settings it is replacing vaginal birth as the norm. It is certainly an attractive option, which eliminates the pain, anxiety and uncertainty as well as the rare unpredictable calamities of labour. It is difficult to balance these tangible short-term benefits with the less tangible but increasingly evident longer-term adverse effects, particularly on neonatal mortality, childhood health and development [6] and maternal postpartum depression [7] and mortality (about 1% for caesarean deliveries in Africa).

Among the many unnecessary ways in which we make birth difficult for women in our care, perhaps the most profoundly damaging is to isolate them from the comfort and support which in a non-clinical setting they would enjoy from those close to them during this most demanding and meaningful of Life's experiences. Apart from the sheer inhumanity of policies excluding supportive companions from labour wards, randomized trials have shown that companionship is associated with 31% reduction in a negative birth experience, 38% reduction in low Apgar scores and 25% reduction in caesarean sections [5]. Women randomly allocated to supportive companionship from a mature volunteer companion from the local community experienced significantly less postnatal depression and anxiety and were significantly more likely to regard themselves as the person best able to care for their baby, to bring their baby with them to postnatal appointments, and to breast-feed [8, 9] After decades of knowing better, the painfully slow progress in reversing labour care policies which compound the distress of women during labour is an indictment of our collective priorities as caregivers.

One way to achieve improvements in labour care is to implement use of the new WHO ‘Labour Care Guide’, which requires health care providers to explicitly document supportive care activities during labour such as companionship, oral hydration, pain relief and avoiding the supine position, and to document action to correct lapses, as well as de-emphasizing the erroneous expectation that all normal labours progress at the average rate of 1 cm per hour cervical dilation [10].

## Contributors

G Justus Hofmeyr is the sole author of this editorial.

## Funding

No funding from an external source supported the publication of this editorial.

## Provenance and peer review

This editorial was commissioned and not externally peer reviewed.

## Conflict of interest statement

The author has an interest in a re-usable postpartum blood loss monitoring tray.
